# Supplementary figures and images for: Integrated metabolomics and bioactivity analysis of new chrysanthemum cultivar petals: Insights into eye-protecting agents
Source: PLoS One. 2026 Feb 9;21(2):e0340052. doi: 10.1371/journal.pone.0340052 (PMC12885252; doi:10.1371/journal.pone.0340052)

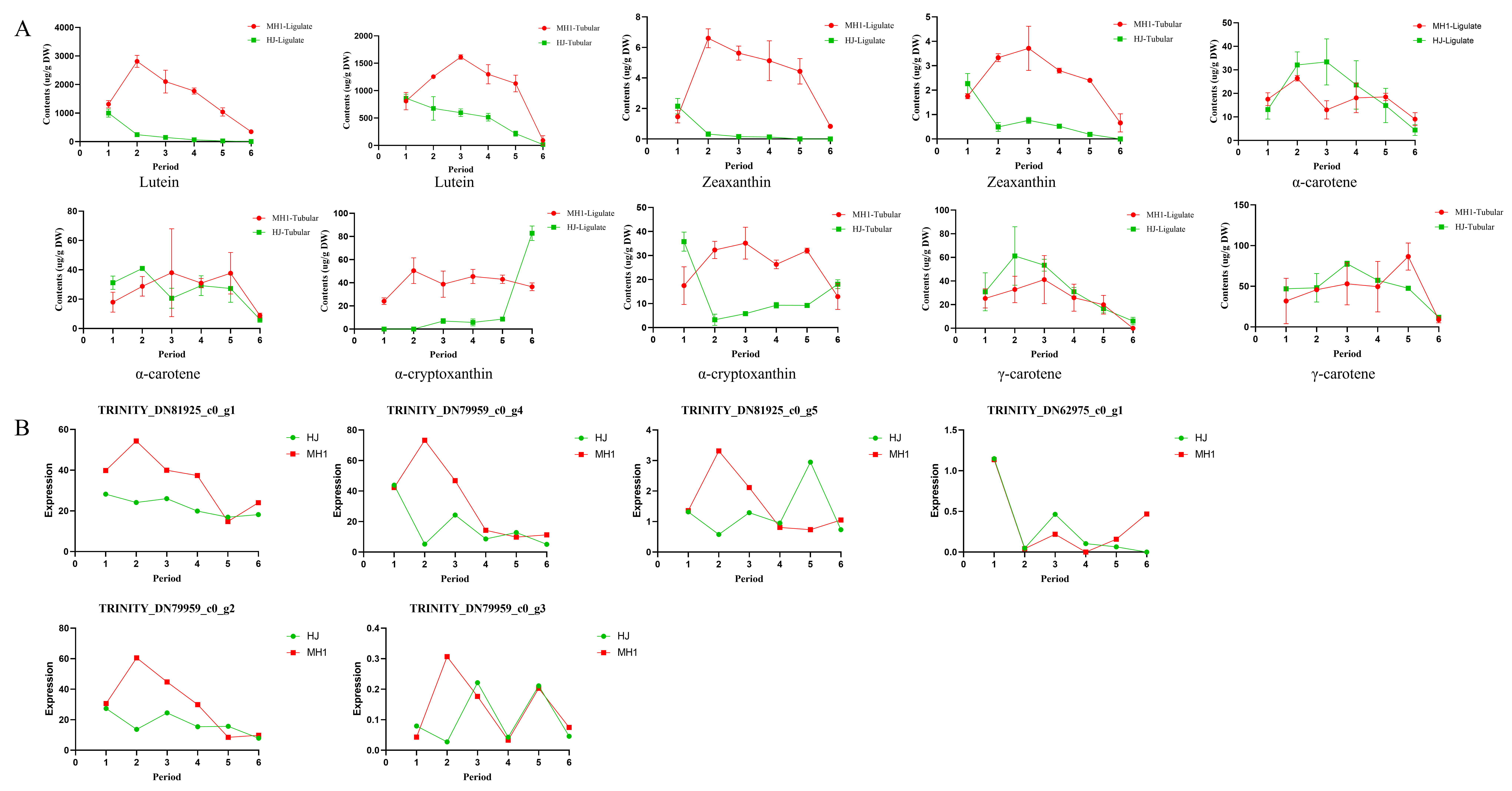

Supplement: S1 Fig — (TIF) [file pone.0340052.s001.tif]
