# Supplementary material for: Integrated metabolomics and bioactivity analysis of new chrysanthemum cultivar petals: Insights into eye-protecting agents
Source: PLoS One. 2026 Feb 9;21(2):e0340052. doi: 10.1371/journal.pone.0340052 (PMC12885252; doi:10.1371/journal.pone.0340052)
Supplement: Attachment — Submitted filename: Response to editor and reviewers.docx [file pone.0340052.s005.docx]

Response to editor and reviewers’ comments:

Subject: Response to editor and reviewers’ comments

Ref: PONE-D-25-34406

Title: Integrated Metabolomics and Bioactivity Analysis of new Chrysanthemum cultivar Petals: Insights into Eye-Protecting Agents

Dear Editor Fahrul Nurkolis and Reviewers:

We sincerely appreciate you and the reviewers for taking the time to review our paper and for providing valuable comments. We have carefully revised each comment according to the suggestions from reviewers. We sincerely appreciate your valuable feedback, which has greatly enhanced the quality of our work. We hope that the revisions meet your expectations and thank you for your time and consideration.

Below, we provide point-by-point responses. All modifications in the manuscript have been highlighted.

1) Thank you for your email and for bringing the change in the author list to our attention. We sincerely apologize for the oversight and any inconvenience it may have caused. The alteration was unintentional and resulted from an operational error while we were updating the submission system. During the process of designating Dr. Gangqiang Dong as a co-corresponding author- a role we intended to add to acknowledge his pivotal contributions to this new variety-we inadvertently deleted the entire author list and had to re-enter it manually. We would like to assure you that the sequence of authors has not been altered in any way.
2) We has also revised our manuscript at the detail on (1) methods of sacrifice, (2) methods of anethesia and/or analgesia, and (3) efforts to alleviate suffering.

Reviewer #1: The manuscript 'Integrated Metabolomics and Bioactivity Analysis of new Chrysanthemum cultivar Petals: Insights into Eye-Protecting Agents' reports a very thorough, well-conducted analysis of the molecular content and synthesis in Chrysanthemum flowers. As such, it is no doubt that this part is worth publishing, eventually in its present form.
Not being an expert in assessing the bioactivity of molecular compounds, I cannot judge the part of the paper concerning the ests in Zebrafish. However, again, the metabolomic and transcrptomic part of this paper is certianly valuable enough to be published.

Reviewer #2: Comments on the article- PONE-D-25-34406ss
Minor comments

Line 21 (Abstract): should be ‘Economical’ and not ‘Economically’

Answer: it was revised in manuscript Line 21.
Line 38, 39 (Introduction): Check the grammar

Answer: it was revised in Introduction Line 38, 39.
Line 92 (Materials n Methods): check the sentence

Answer:it was revised in Materials and Methods Line 92.

Line 127 (section 2.2.1): Check the sentence

Answer:it was revised in section 2.2.1 Line 127
Line 160-162 (section 2.4): Please specify if there are two mobile phases and hence rectify the sentence

Answer:we confirm that two mobile phases were employed in this study, and the corresponding sentence (Lines 160-162) has been rectified to clearly specify this information.
Section 2.9 (Statistical analysis): The statistical tests appear to be applied appropriately. However, the rationale or the specific purpose behind each test could be clarified better; otherwise, readers less familiar with metabolomics would find this, a bit difficult to comprehend.

Answer:we have added discussion
Table 1: It is unclear as to why the compound ‘Acacetin-7-O-β-D-rutinoside’ is highlighted out of all other compounds in table 1. Mentioning the reason in the table caption or in a relevant section would give the table better clarity.

Answer:the highlighting of ‘Acacetin-7-O-β-D-rutinoside’ in Table 1 was an unintended formatting error during manuscript preparation. We have removed the unnecessary highlighting to ensure consistency and clarity of the table.
Results- The results are presented in detail, but in several cases their broader biological or functional implications are not clearly explained. A more explicit interpretation of what these findings mean in the context of the study objectives would greatly benefit readers. In short a ‘Discussion’ or ‘Interpretation’ section would further strengthen the manuscript.

Answer: we have supplemented a systematic interpretation of the biological significance and functional value of the research findings.
.However, the rationale for certain analyses and the broader implications of the results are not always explained in sufficient detail, which may make the manuscript less accessible to readers outside the immediate field. Providing clearer interpretation and including a dedicated discussion section would enhance the clarity, impact, and overall readability of the study.

Answer: we have added discussion

Journal Requirements:

When submitting your revision, we need you to address these additional requirements.

1.Please ensure that your manuscript meets PLOS ONE's style requirements, including those for file naming. The PLOS ONE style templates can be found at

https://journals.plos.org/plosone/s/file?id=wjVg/PLOSOne_formatting_sample_main_body.pdf and

<https://journals.plos.org/plosone/s/file?id=ba62/PLOSOne_formatting_sample_title_authors_affiliations.pdf>

Answer:we ensure that our manuscript meets PLOS ONE's style requirements

2. To comply with PLOS One submissions requirements, in your Methods section, please provide additional information regarding the experiments involving animals and ensure you have included details on (1) methods of sacrifice, (2) methods of anesthesia and/or analgesia, and (3) efforts to alleviate suffering.

Answer:we have provided additional information regarding the experiments involving animals in our Methods section.

3. Please note that funding information should not appear in any section or other areas of your manuscript. We will only publish funding information present in the Funding Statement section of the online submission form. Please remove any funding-related text from the manuscript.

Answer：the funding information present only in the funding statement section.

4. We note that the grant information you provided in the ‘Funding Information’ and ‘Financial Disclosure’ sections do not match.

When you resubmit, please ensure that you provide the correct grant numbers for the awards you received for your study in the ‘Funding Information’ section.

Answer:We have verified the grant information and corrected the inconsistencies between the ‘Funding Information’ and ‘Financial Disclosure’ sections.

5. We note that your Data Availability Statement is currently as follows: All relevant data are within the manuscript and in Supporting Information files.

Please confirm at this time whether or not your submission contains all raw data required to replicate the results of your study. Authors must share the “minimal data set” for their submission. PLOS defines the minimal data set to consist of the data required to replicate all study findings reported in the article, as well as related metadata and methods (https://journals.plos.org/plosone/s/data-availability#loc-minimal-data-set-definition).

For example, authors should submit the following data:

- The values behind the means, standard deviations and other measures reported;

- The values used to build graphs;

- The points extracted from images for analysis.

Authors do not need to submit their entire data set if only a portion of the data was used in the reported study.

If your submission does not contain these data, please either upload them as Supporting Information files or deposit them to a stable, public repository and provide us with the relevant URLs, DOIs, or accession numbers. For a list of recommended repositories, please see https://journals.plos.org/plosone/s/recommended-repositories.

If there are ethical or legal restrictions on sharing a de-identified data set, please explain them in detail (e.g., data contain potentially sensitive information, data are owned by a third-party organization, etc.) and who has imposed them (e.g., an ethics committee). Please also provide contact information for a data access committee, ethics committee, or other institutional body to which data requests may be sent. If data are owned by a third party, please indicate how others may request data access.

Answer:The original data has been uploaded as supplementary materials.

6. If the reviewer comments include a recommendation to cite specific previously published works, please review and evaluate these publications to determine whether they are relevant and should be cited. There is no requirement to cite these works unless the editor has indicated otherwise. 

I appreciate you taking time to review this revision and look forward to hearing from you soon.

Yours sincerely,

Associate Professor Dr Jing Peng

Institute of Plant Protection,

Hunan Academy of Agricultural Sciences
